# Lack of maintenance of motorway fences works against their intended purpose with potential negative impacts on protected species

**DOI:** 10.1038/s41598-020-57767-4

**Published:** 2020-01-21

**Authors:** Miguel A. Farfán, Julia E. Fa, Adrián Martín-Taboada, José María García-Carrasco, Jesús Duarte

**Affiliations:** 1 Departamento de Biología Animal, Facultad de Ciencias, Universidad de Málaga, Málaga, Spain; 2 Department of Natural Sciences, Manchester Metropolitan University, Manchester, M15 6BH UK; 3 Ofitecma Marbella SL, Avda. Ramón y Cajal 17, 29601 Marbella Málaga, Spain

**Keywords:** Conservation biology, Urban ecology

## Abstract

Linear infrastructure intrusions into natural ecosystems, such as motorways and high-speed railways, causes direct loss of habitat but also impacts fauna through collisions. Wildlife road mortality is well documented and extensive conservation legislation exists in many countries to minimise the negative impact of these infrastructures. However, although these measures are implemented because of legislation, these structures are often not adequately maintained. Here we present data on the functionality of perimeter fences along two motorways in Malaga province (southern Spain) erected to prevent collisions with the common chameleon (*Chamaeleo chamaeleon*). We sampled the fences along the 14 km of the two motorways included in the 17 1 × 1 km squares of the study area. Our results show that the reptile fence is permeable throughout at those points where the metal sheeting was absent and where the vegetation had overgrown around the fence, hence allowing chameleons to cross. Given our results, we conclude that this situation is likely to be similar in other regions of Spain and in other countries. This is because construction/concessionary companies do not consider the environmental impact of construction projects in the medium and long term, and environmental authorities do not ensure that companies comply with the legislation.

## Introduction

Cities, states and metropolitan areas throughout the world share an underlying need for modern, efficient and reliable infrastructure. Man-made linear infrastructure such as roads and motorways cause disruptions of terrestrial ecosystems and wildlife populations, such as habitat loss and fragmentation, spread of invasive alien species as well as animal injury and mortality e.g., roadkill^1–3^.

The ecological effects of road infrastructure intrusions have been extensively investigated to understand the causes of impact and how it can be mitigated^1,4^. Road mortality rarely affects population viability^5^ of the more abundant species with high reproductive rates^6^, but it can be detrimental for species of low population densities and reproductive rates (reviewed by^7^). The sensitivity of some wildlife populations as well as the danger to people posed by wildlife‐vehicle collisions has encouraged the field of road ecology and mitigation efforts (reviewed by^4,5,8^). In the case of roads, mitigation often consists of exclusion fencing (to prevent animals from accessing the road) coupled with crossing structures^9^.

Wildlife road mortality in European countries can be high. As many as 27 million birds have been estimated to be killed annually on roads in some European countries^10^, and in Spain alone at least 10 million vertebrate deaths are caused by vehicle collisions^11^. These numbers, although significant, are likely to be an underestimate given that assessments of vertebrate road mortality in other countries, such as the USA, can be as high as 1 million individuals per day^12^. As a result, European environmental legislation now insists that an Environmental Impact Assessment (EIA) is completed before proposals for a motorway or railway project are allowed to start^13–15^. Depending on the EIA, authorities may insist that the construction company implement a variety of mitigation measures to protect wildlife^16,17^. For reptiles and amphibians, these measures must include searching and picking up all individuals before the start of works^15^. Additionally, to prevent animals crossing the road or track, fences are to be erected once the infrastructure is completed^18–20^. To guarantee fence effectiveness, these are to be designed according to the characteristics and requirements of the target species, implemented correctly, and must be regularly maintained^20–22^.

In this study, we examine the success of exclusion structures designed for and installed around motorways to avoid road mortality of the common chameleon (*Chamaeleo chamaeleon*) in southern Spain. In Europe, the species is found only within a small distribution range in southern Spain and Portugal. Although the species may have been introduced to Iberian Peninsula from northern Africa in prehistoric or and historical times^23^, since the Spanish populations are morphologically indistinguishable from those of northern Africa^24^, individuals of the species are not allowed to be killed nor their breeding sites or resting places disturbed. The common chameleon is included in Annex IV of the EU Habitat and Species Directive (92/43/CE) as a species of interest and is strictly protected in Annex II of the Bern Convention. In Spain, mortality caused by collision with vehicles is one of the main threats to the species^25–27^.

## Material and Methods

### Study area

**Figure 1:**
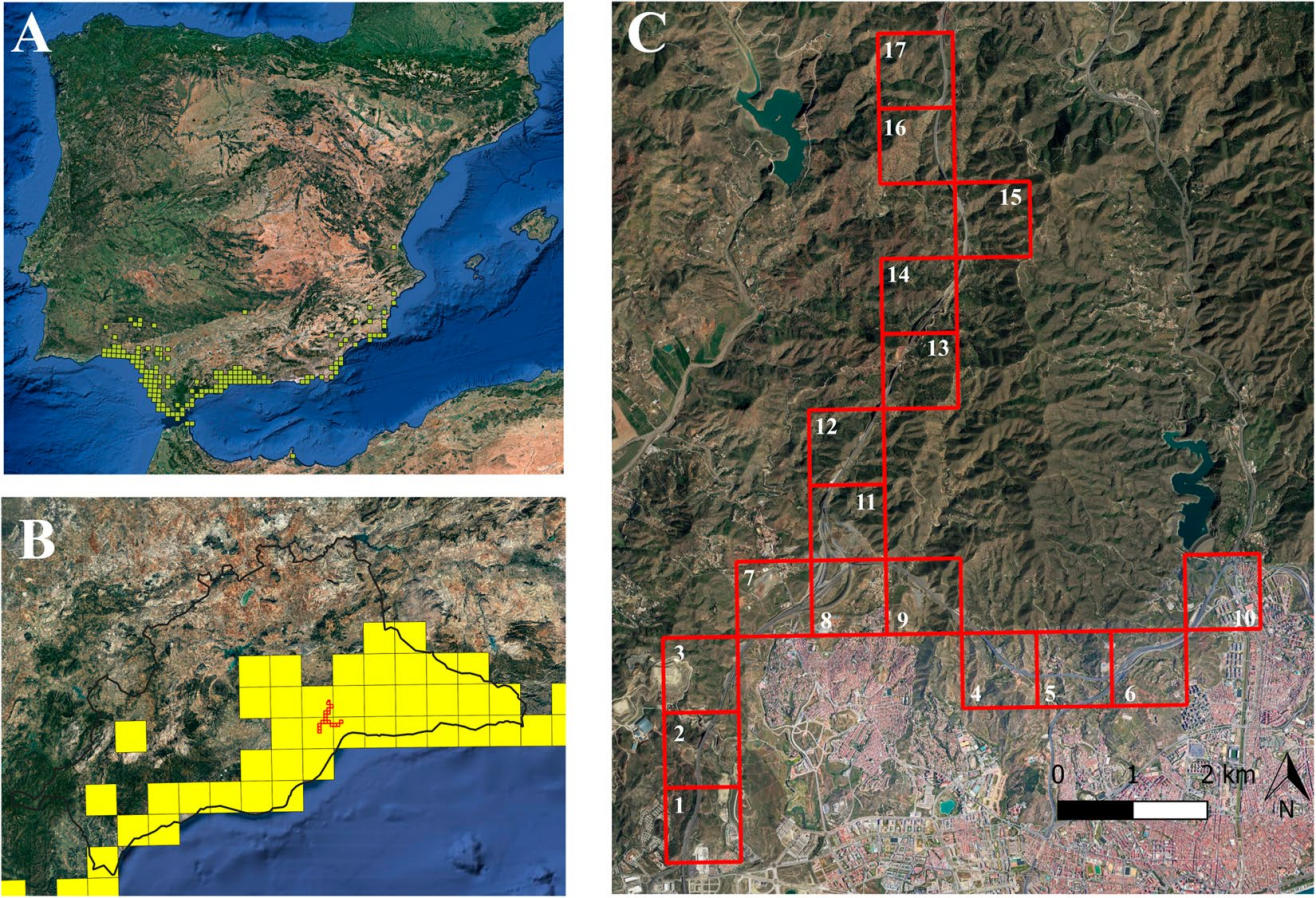
(A) Iberian Peninsula with common chameleon distribution in 10 × 10 km green squares (taken from http://siare.herpetologica.es/bdh/distribucion). (**B**) Delimitation of Malaga province (black line) with common chameleon distribution in 10 × 10 km yellow squares, and the study area in 1 × 1 km red squares. (**C**) Study area in 1 × 1 km red squares. Created with ArcGIS 10.6 (source: Esri, DigitalGlobe, GeoEye, Earthstar, Geographics, CNES/Airbus, DS, USDO, USGS, AeroGRID, IGN, and the GIS User Community). The numbers of each square correspond to the numbers in the “Square” field of Table 1.

Our study area is located within the main distribution zone of the common chameleon^25^. This area is considered as one of the populations experiencing significant declines in the south of Spain^28^. We focused on two motorways in the north of the city of Malaga - AP-46 “Las Pedrizas” (24.5 km) and A-7 “Ronda Oeste de Málaga” (23 km); at its southernmost point the AP-46 connects with the A-7 (Fig. 1). These motorways came into operation in 2010–2011. The AP-46 has a N-S orientation and together with the A-45 road that runs parallel to it, are the main access roads to Malaga for the cities in central and northern Spain. The A-7 has an E-W direction and connects Malaga with the coastal cities to the east and west of Spain (Fig. 1).

In this study, we investigated the following kilometre points (kp): 1) AP-46, kp 24 (S)-kp 17 (N). 2) A-7, kp 241 (E)-kp 234 (W). We divided the length of these sections into 1 × 1 km squares, the territorial units used in our analyses. The landscape surrounding the motorways is open, with varying elevations. The area has a Mediterranean climate with an average temperature of 12.3 °C in January and 25.6 °C in August, and an annual precipitation of 520 mm^29^. The dominant vegetation is composed of crops, mainly olive trees (*Olea europaea*) and almond trees (*Prunus amygdalus*), and scrubland constituted by Retama broom (*Lygos sphaerocarpa*), European fan palm (*Chamaerops humilis*), rosemary (*Rosmarinus officinalis*) and rock rose bushes (*Cistus spp*.).

### Fence surveys

**Figure 2:**
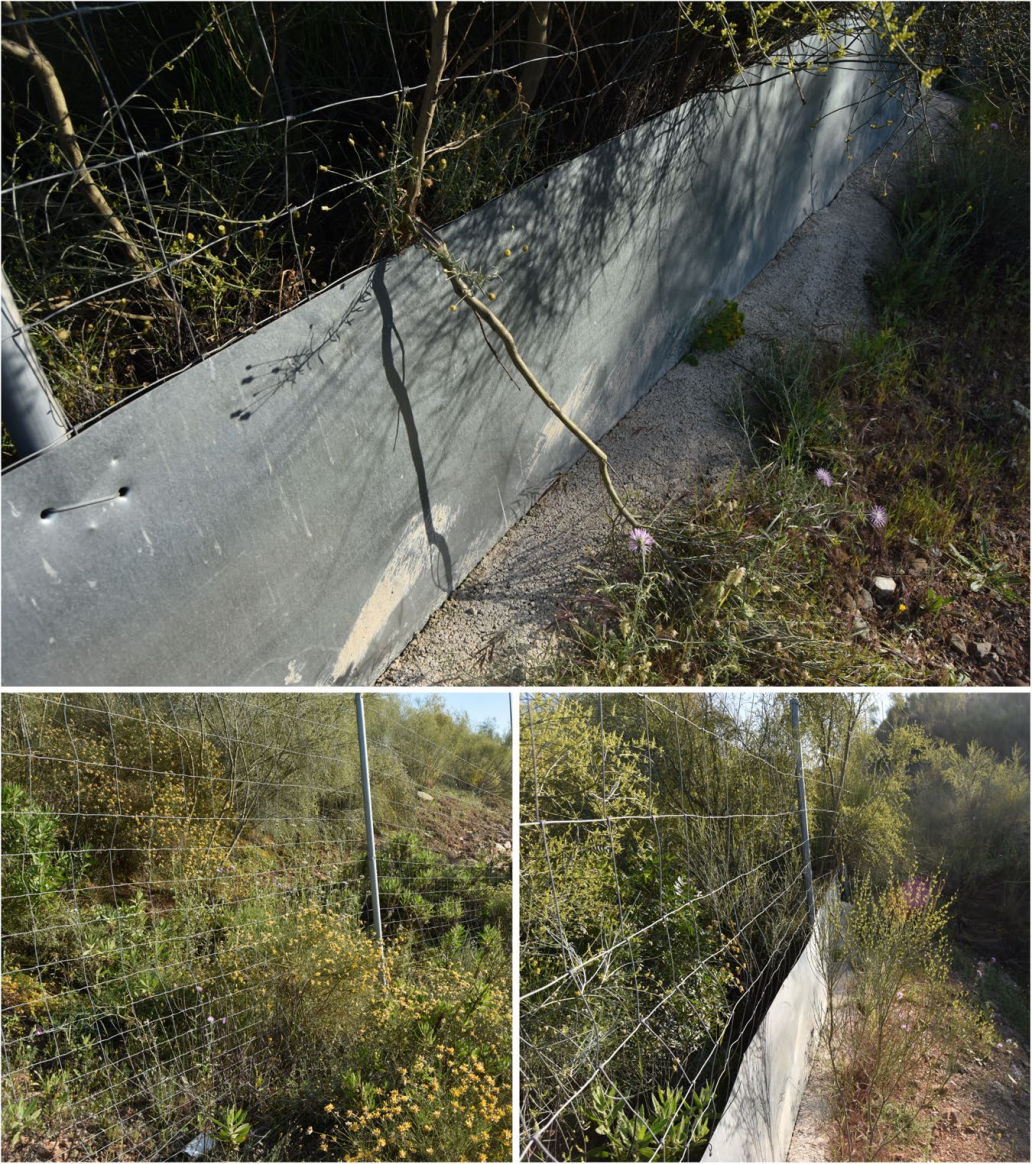
Top image: reptile fence. Left image: chain link fence without metal sheet. Right image: reptile fence with vegetation around it.

Exclusion fences are found continuously along the AP-46 and A-7 motorways. To exclude reptiles from highways, an aluminum metal sheet extending 80 cm above and 10 cm below-ground was affixed to the base of a 2 m tall chain-link fence to exclude large ungulates (Fig. 2).

During June 2019 we inspected the fences along the 14 km of the two motorways included in the 17 1 × 1 km squares of the study area. We searched for breaks in the continuity of the reptile fences. We classified these interruptions as: a) the absence of the metal sheet and b) the presence of vegetation growing around the fence (Fig. 2). Fences were inspected by two observers from a car driven by a separate person at constant speed of 80 km/h along the motorways (see^30^). Observers recorded the location of breaks in the reptile fence using the Mapas de España (6.5) mobile application software^31^. We recorded all fence breaks on both sides of the two motorways. All locations were downloaded onto a computer and processed using ArcGIS 10.6. We calculated the percentage of 1 × 1 km squares of the study area where the motorways had anomalies on the reptile fence.

## Results and Discussion

We detected anomalies in at least one point within the road sections included in all 1 × 1 km squares of the study area. For both the percentage of squares in which the fence was permeable due to vegetation growing around it, and as a result of the absence of metal sheet portions was the same (94.1%) (Table 1). Absence of the metal sheet as well as vegetation growing over it was found in 88% of squares.

**Table 1:** Types of anomalies detected in the 1 × 1 km squares of the study area.

Square	Metal sheet	Vegetation	Both
1	1	1	1
2	1	1	1
3	1	1	1
4	1	1	1
5	1	1	1
6	1	1	1
7	1	1	1
8	1	1	1
9	1	1	1
10	1	0	0
11	1	1	1
12	1	1	1
13	1	1	1
14	1	1	1
15	1	1	1
16	1	1	1
17	0	1	0
N	**16**	**16**	**15**
%	**94.1**	**94.1**	**88.2**

Total squares of the study area = 17 (see Fig. 1). 0: reptile fence without anomaly; 1: reptile fence with anomaly.

Although there is published evidence to show that mitigation measures along roads can reduce animal mortality^19,32^, relatively few studies have investigated their effectiveness^6^, especially over the longer term^33^. Our study, arguably the first one in Spain demonstrates that although road mortality mitigation measures can be effective^5,34^ their impact can easily be nullified without adequate follow-up maintenance. We show that in all 1 × 1 km squares, all fences inspected by us were permeable, suggesting that these barriers posed a threat to the endangered species for which they were meant to protect. If this is the case along the entire length of fences for all motorways within the distribution range of the chameleon in southern Spain, the impact of the lack of fence maintenance could be devastating for the species.

Understanding of the environmental impact associated with the expansion of linear infrastructures (roads or railways) has proliferated in recent decades^35^. Parallel to this, in many countries specific environmental legislation has emerged, aimed at mitigating or eliminating the negative effect of infrastructures on wildlife. For example, the approval of an EIA by the competent environmental authorities is now required by construction companies before starting any infrastructure project. In many cases, the implementation of mitigation measures is also required; the latter often adding greater expense to the construction projects. As examples, the USA government spent 94 million dollars on road mitigation measures between 1992–2008^36^ and in the Netherlands, a total of 70 million euros (10% of road project budget) were spent by the government to counteract the negative impact of a 42-km highway on wildlife^37^.

Overall, motorway construction/concessionary companies are aware of their legal obligations and often show good disposition to help minimize road/wildlife conflict^21^. Moreover, construction/concessionary companies are also aware that the installed structure to mitigate impacts deteriorate with the passage of time and suffer damage compromising their effectiveness, thus adequate maintenance is fundamental, with regular inspections and immediate repairs. Despite this, construction/concessionary companies lack standardized protocols for evaluating the long-term effectiveness of the mitigation measures implemented. The result is that little attention is still given to ensure that environmental impacts are lessened. As we show in our study, if no attention is placed in maintaining the effectiveness of road mortality mitigation measures, we argue here that this is effectively the abandonment of responsibilities by competent authorities in environmental conservation matters. Thus, to achieve adequate environmental integration of large infrastructure projects, it is essential that environmental authorities enforce environmental legislation that obliges construction companies not just to implement mitigation measures but to maintain these over the entire lifetime of the infrastructures^21^. Construction/concessionary companies must assume that mitigation measures are more than specific actions taken to satisfy legislation, as it is a means to develop infrastructures that really reduce the impact that infrastructures have on wildlife^19^. This implies that construction/concessionary companies have to bear the costs of implementing mitigation measures and to maintain these in good condition over time.
